# Antiretroviral therapy, perceived health, and fertility decision-making among people living with HIV: a qualitative study at a district antiretroviral therapy clinic in the Ashanti Region, Ghana

**DOI:** 10.3389/frph.2026.1914707

**Published:** 2026-09-29

**Authors:** Priscilla Asantewaa Boadi, Victor Luckyboy Dzramado

**Affiliations:** 1 St. Michael Nursing and Midwifery Training College, Kumasi, Ghana; 2 Department of Biostatistics, Cape Coast Teaching Hospital, Cape Coast, Ghana

**Keywords:** antiretroviral therapy, fertility intentions, Ghana, HIV, prevention of mother-to-child transmission, qualitative research, reflexive thematic analysis, reproductive counselling

## Abstract

Antiretroviral therapy has changed what parenthood can mean for people living with HIV, yet little is known about how patients at district-level clinics in West Africa themselves connect treatment, bodily health, and decisions about childbearing. This study explored how people living with HIV attending one district antiretroviral therapy clinic in Ghana's Ashanti Region described the place of treatment in their reproductive thinking. A qualitative interview study analysed using reflexive thematic analysis informed by an interpretive phenomenological orientation. Twelve adults receiving antiretroviral therapy at the St. Michael's Hospital clinic in Pramso took part in semi-structured interviews conducted in English or Twi between August and October 2024. Interviews were audio-recorded, transcribed, and coded inductively, with the Theory of Planned Behaviour applied interpretively after coding. Four participants described treatment access and clinic services as adequate. Within these accounts, one participant linked daily medication-taking to a felt state of bodily wellness, and one reported believing that medication would not harm a baby; no participant explicitly connected bodily wellness to a decision about childbearing. Transmission concern was more widely expressed, and several participants relayed provider messages that a baby could be protected, with one woman also reporting having been told how to conceive without infecting her partner. Fertility positions were heterogeneous: some deferred decisions, some limited further births after completing their families, one woman described pressure from her husband to have another child, and the one man whose account addressed this topic framed his position economically rather than around transmission. Emotional uncertainty persisted alongside accurate prevention information. No participant referred to viral load results, undetectable status, or viral load monitoring, and the interview guide did not ask about these. Reproductive positions at this clinic were shaped by parity, partnership, economic circumstance, and emotion, with HIV status carrying variable weight. The data show that treatment access was valued but do not establish that perceived health drove reproductive intentions, and because viral suppression was neither verified nor discussed, claims that suppression shapes fertility decisions are not supported. Findings are specific to this setting and indicate scope for counselling that pairs biomedical information with emotional and individualised support.

## 1 Introduction

The scale-up of antiretroviral therapy has altered the life course of people living with HIV, converting a once-fatal infection into a long-term condition compatible with work, partnership, and family life (1). Adults who start treatment early and remain in care can now approach near-normal life expectancy in well-resourced cohorts (2), and global guidance treats sustained therapy as central to both individual health and onward prevention (3). Ghana has widened access to free antiretroviral therapy and improved testing coverage over successive programme cycles, while retention, adherence, and durable suppression remain live challenges (4, 5).

Adherence in Ghana is measurably imperfect. A national systematic review and meta-analysis found pooled optimal adherence below the level conventionally required for reliable suppression, with the lowest adherence among younger patients (6). Laboratory outcomes reflect this. Among 7,199 adults in Ghana's western region, viral load suppression was achieved by 5,465 patients (75.91%), while 1,734 (24.09%) were unsuppressed (7). A Ghanaian cohort measured at three points reported that the proportion with viral load below 50 copies per millilitre rose from 74% at baseline to 88% at eighteen months, with lower educational attainment associated with viral rebound (8). Adherence is therefore a daily accomplishment for patients, not merely a clinical indicator.

Clinically, sustained suppression carries a prevention benefit of the first importance. The PARTNER study recorded no phylogenetically linked transmissions among serodifferent couples across tens of thousands of episodes of condomless sex when the partner with HIV was suppressed (9), HPTN 052 showed a 93% reduction in linked transmission with early treatment (10), and subsequent studies confirmed the absence of linked transmissions among suppressed serodifferent gay couples (11, 12). This evidence base underpins current safer conception guidance. Whether and how it reaches patients at district level in West Africa, however, is a separate empirical question, and one this study is positioned to address only from the patient side.

Fertility desires among people living with HIV remain substantial. Expanding treatment access has accompanied sustained reproductive aspiration across the region (13, 14). In Soweto, South Africa, 47% of 442 patients on treatment expressed a desire for children, with reasons including having no biological child, partner wishes, and feeling healthy after starting therapy; absence of a biological child was strongly associated with fertility desire (AOR 5.06, 95% CI 2.11 to 12.1) (15). Desires are also gendered. In a mixed-methods study in Nairobi informal settlements, the adjusted odds of desiring a child were more than four times higher among men than women (OR 4.17, 95% CI 2.46 to 7.07) (16), and comparable patterns appear in Ethiopian clinic populations (17). Reproductive intentions are shaped by transmission fears, health concerns, partner dynamics, stigma, and economic constraint, which is why integration of reproductive services into HIV care has long been recommended (18, 19). Safer conception provision across sub-Saharan Africa nevertheless remains uneven, and providers frequently do not initiate discussion of fertility goals (20, 21). Counselling for women living with HIV in low and middle income settings improves knowledge and service use, though coverage varies (22). In Ghana, pronatalist and patriarchal expectations add social weight to women's reproductive lives (23, 24).

The Theory of Planned Behaviour, which holds that attitudes, subjective norms, and perceived behavioural control jointly shape intention, offers one lens for interpreting such accounts, and has been applied to adherence among people living with HIV (25, 26). In the present study this theory was not used to design the interview guide or to construct the coding frame; it was applied interpretively after inductive coding, and this sequence is reported explicitly in the Methods.

What remains poorly described is how patients at a district clinic in West Africa themselves talk about the relationship between taking medication, feeling well, and thinking about children. This study therefore explored, through the accounts of twelve adults attending one antiretroviral therapy clinic in Ghana's Ashanti Region, the place of treatment in reproductive thinking. The aim is descriptive and interpretive rather than explanatory, and the findings are offered as transferable insight into one setting rather than as a general account of people living with HIV in Ghana.

## 2 Materials and methods

Reporting follows the Consolidated Criteria for Reporting Qualitative Research (COREQ), and a completed 32-item checklist accompanies this submission as Supplementary Material S2 (27).

### 2.1 Design and methodological orientation

This was a qualitative interview study. The analytic approach was reflexive thematic analysis informed by an interpretive phenomenological orientation. Interpretive phenomenology guided the study's interest in how participants made sense of living with HIV while considering children, and it shaped the emphasis on meaning in social and clinical context (28, 29). The analytic procedure implemented was thematic: line-by-line inductive coding, grouping of codes into categories, and construction of themes across cases (30). The study did not implement the idiographic, case-by-case analytic sequence characteristic of interpretative phenomenological analysis, and it is not presented as such.

### 2.2 Research team, positionality, and reflexivity

The study formed the master's research of the first author (PAB), a female Ghanaian nurse-midwife and nurse educator based at a midwifery training college in Kumasi, who holds a professional nursing qualification and received training in qualitative interviewing as part of her postgraduate programme. PAB conducted all interviews and performed the initial coding. She was not employed at St. Michael's Hospital, held no clinical role in participants' care, and had no prior relationship with any participant. Participants were told that she was a nurse and postgraduate student conducting research for an academic degree, that she was not part of the clinic team, and that nothing they said would be entered into their medical records or affect their treatment.

The second author (VLD) is a male biostatistician at a teaching hospital who was not present at any interview and had no contact with participants. He contributed to design, analytic discussion, and interpretation. Academic oversight was provided by a thesis supervisor at the awarding university, who was not an author, not a member of clinic staff, and had no clinical relationship with participants, and whose role was limited to discussion of emerging codes and review of the developing thematic structure.

Reflexivity was operationalised in three ways. First, PAB kept a written reflexive journal in which she recorded, after each interview, her emotional reactions, the questions she had found difficult to ask, and instances where she suspected a participant had answered to please her. Second, entries were reviewed against the transcripts during coding, and codes that appeared to originate more in the researcher’s expectation than in participants’ words were revised or removed. Third, PAB’s professional background was treated as an explicit analytic consideration: as a nurse-midwife she began the study assuming that clinical counselling would emerge as the dominant influence on reproductive thinking, and journal entries record her revising this assumption when economic and relational considerations proved more prominent in several accounts.

Two power-related risks were considered throughout. Interviews took place inside the facility where participants receive care, and participants may have felt encouraged to speak favourably about the clinic. The positive service descriptions reported below should be read with that possibility in mind, and are presented as participants’ accounts rather than as service evaluation. Participants may also have overstated their adherence to a nurse interviewer, which is why the analysis reports perceived rather than verified adherence.

### 2.3 Study setting

The study was conducted at St. Michael’s Hospital, a district-level facility at Pramso in the Bosomtwe District of the Ashanti Region, approximately twenty kilometres from Kumasi. The hospital is a Catholic mission facility operating within the Christian Health Association of Ghana network and, like other facilities in that network, delivers government health policy while working within denominational teaching. Catholic institutions constitute the largest denominational bloc within that network and do not provide artificial contraception, referring clients requesting such methods to nearby facilities that do (31). The antiretroviral therapy clinic provides treatment, CD4 and viral load monitoring, management of opportunistic infections, and counselling within Ghana's national programme, which supplies free antiretroviral therapy to eligible patients. Approximately 120 adults were registered at the clinic during the study period. This institutional character is relevant to interpretation and is returned to in the Discussion.

### 2.4 Sampling, recruitment, and sample adequacy

Participants were eligible if they were aged eighteen years or older, were receiving antiretroviral therapy at the clinic, were able to give informed consent, and were at a life stage at which childbearing decisions were relevant to their own circumstances. No upper age limit was applied, and the criterion was framed to accommodate both women and men.

Recruitment took place during routine clinic visits. A senior clinic nurse, who was not a member of the research team, identified patients meeting the criteria from the appointment list and asked whether they were willing to hear about a study. Those who agreed were introduced to PAB in a private room, where she explained the study and sought consent. This arrangement protected patient privacy but carries an acknowledged limitation: patients who attend appointments regularly, and who are comfortable with clinic staff, were more likely to be approached. Patients disengaged from care, or dissatisfied with services, are correspondingly likely to be underrepresented. Formal counts of those approached, assessed as eligible, declining, and enrolled were not maintained, which is a documentation shortcoming reported here rather than reconstructed.

Purposive sampling sought variation in sex, age, marital status, parity, employment, and educational background, and the achieved sample reflects that intention across those dimensions (Table 1). Variation in partner HIV status was not systematically sought and was not consistently recorded, which is a limitation given the relevance of serodifference to conception planning.

**Table 1:** Demographic characteristics of participants.

Participant code	Age band (years)	Sex	Marital status	Education	Employment	Number of children	Children's HIV status (participant-reported)
P1	25–29	Female	Single	Tertiary	Student	0	Not applicable
P2	35–39	Female	Divorced	Secondary	Unemployed	1	Negative
P3	25–29	Female	Married	Tertiary	Employed	0	Not applicable
P4	35–39	Male	Married	Secondary	Employed	2	Negative
P5	40–44	Female	Widowed	None	Unemployed	2	Negative
P6	35–39	Female	Married	Tertiary	Employed	1	Negative
P7	45–49	Male	Married	Tertiary	Employed	6	Negative
P8	25–29	Female	Single	Tertiary	Student	0	Not applicable
P9	45–49	Female	Married	Secondary	Employed	3	Negative
P10	35–39	Female	Single	Tertiary	Employed	3	Negative
P11	25–29	Male	Single	Secondary	Employed	0	Not applicable
P12	30–34	Female	Married	Tertiary	Employed	2	Negative

Recruitment continued alongside preliminary analysis. PAB and the academic supervisor jointly judged, after twelve interviews, that later interviews were producing accounts that echoed earlier ones on the core topics of treatment access, transmission concern, and counselling experience, without generating new categories. This was a contemporaneous judgement about code-level adequacy on those topics, and it is not presented as demonstrated saturation.

Two features of the achieved dataset qualify that judgement further, and are stated here rather than left to the reader to infer. First, the material available for four participants did not speak substantively to any reported theme, so the effective analytic sample was eight rather than twelve. Second, substantive fertility-related content was available from only one of the three men. Saturation cannot therefore be claimed for the dataset as a whole, and it certainly cannot be claimed for men's accounts or for the mechanics of fertility decision-making, where the material is thinnest. What the twelve interviews support is a description of the range of positions present at this clinic, not an exhaustive account of them.

Information power offers a framework for appraising this achieved sample: the sample was narrowly specific, the interviews were focused on a single well-defined topic, and cross-case thematic rather than idiographic analysis was undertaken, all of which reduce the number of participants required (32–34). This framework was applied retrospectively, as a way of interpreting the sample obtained. It did not guide the original sampling strategy, and it did not inform the decision to stop recruiting.

### 2.5 Data collection

Semi-structured individual interviews were conducted in a private room at the hospital between 4 August and 5 October 2024, and lasted between forty-five and ninety minutes. The guide, developed from the study objectives and refined with the academic supervisor, covered time since diagnosis, experience of living with HIV, current treatment status, experience of health services, existing children and their HIV status, current thoughts about having more children, reasons for wanting or not wanting children, the influence of HIV status on those thoughts, factors considered most important, how providers addressed fertility concerns, counselling received, barriers encountered, and how those barriers affected decision-making. Probes invited elaboration and examples. The guide was not formally pilot tested.

Two features of the guide are important for interpreting the findings. It did not ask about viral load results, undetectable status, or viral load monitoring, and it did not ask separately about contraception, child spacing, or partner participation in decisions. Consequently the dataset speaks to treatment-taking, perceived health, and counselling experience far more directly than it speaks to viral suppression or to the mechanics of joint decision-making, and the analysis has been bounded accordingly.

Interviews were audio-recorded with permission. Field notes recorded setting, demeanour, and interviewer impressions. These notes were used to contextualise transcript readings and to inform reflexive journaling; they are not treated as an independent data source, and the term triangulation is not applied to them, since notes and transcript derive from the same encounter.

### 2.6 Language, transcription, and translation

Participants chose the interview language. Of the twelve interviews, the majority were conducted in Twi, the dominant language of the study area, with the remainder in English or in a mixture of the two. PAB is a first-language Twi speaker and also fluent in English, and she conducted all interviews without an interpreter. Twi recordings were transcribed directly into English by PAB while listening to the audio, rather than being transcribed first in Twi and then translated as a separate step. Independent back-translation was not performed. Where a Twi expression had no direct English equivalent, PAB rendered the sense of the phrase and recorded the original wording in her notes for discussion with the supervisor.

This procedure has consequences that readers should weigh. Because transcription and translation were performed in a single step by one bilingual researcher, and because no independent linguistic check was applied, the published quotations are best understood as that researcher's English renderings of Twi speech rather than as verbatim transcription in the original language. Quotations from interviews conducted in English are closer to direct speech. In preparing quotations for publication, grammar and syntax were left unaltered, so that non-standard English constructions in the excerpts reflect either the participant's own English or the translator's close rendering. Square brackets mark editorial insertions and ellipses mark omitted speech.

### 2.7 Analysis

Transcripts were read repeatedly alongside the audio. Coding was manual, using printed transcripts, marginal annotation, and a spreadsheet register of codes; no qualitative analysis software was used. Codes were generated inductively from participants' language, with no prior coding frame and no theoretical categories imposed at this stage. Related codes were then grouped into categories, and categories were developed into themes describing patterns across the dataset. The hierarchy used in this report is: codes describe segments of talk; sub-themes group codes addressing one aspect of experience; themes group sub-themes. The full codebook of twenty-three inductive codes and the mapping of codes to sub-themes and themes are provided in Supplementary Material S1.

Movement between individual accounts and cross-case themes was achieved by coding each transcript in full before comparison, then reviewing each candidate theme against every transcript to check that it was grounded in more than one or two accounts and to identify cases that did not fit. Accounts that diverged from developing themes were retained and are reported below rather than smoothed away. Candidate sub-themes that a full check against the transcripts did not support were removed rather than retained on thin evidence, and the four themes reported here are those the data sustain.

The Theory of Planned Behaviour did not inform the interview guide or the coding frame. It was introduced only at the interpretation stage, as one available way of reading the finished themes, and its application is confined to the Discussion.

Because the sample is twelve, quantifying language is avoided in favour of explicit counts, and any statement about how many participants expressed a view is traceable to the excerpt tables and to Table 2.

**Table 2:** Participant contribution by theme.

Theme	Contributing participants	Number
Theme 1: Treatment access and service appraisal	P7, P8, P9, P10	4
Theme 2: Transmission concern and reported prevention information	P1, P2, P6, P8, P9, P10	6
Theme 3: Reproductive counselling as experienced by patients	P1, P2, P3, P7, P8	5
Theme 4: Heterogeneity of fertility positions	P1, P2, P3, P6, P7, P8, P9, P10	8

### 2.8 Ethics statement

Ethical approval was granted by the Committee on Human Research, Publication and Ethics at the Kwame Nkrumah University of Science and Technology, College of Health Sciences, School of Dentistry (reference CHRPE/AP/569/24), valid from 8 July 2024 to 7 July 2025. Data collection began on 4 August 2024, after approval took effect. An introductory letter was issued by the College of Health Sciences (reference NURS/INTRO/Vol. 1, 7 March 2024). Institutional permission, as distinct from research ethics approval, was granted by the Catholic Health Service Trust-Ghana (reference SMH/RA.007/24, 6 June 2024) and by the administration of St. Michael's Hospital. The approved protocol addressed fertility intentions among people living with HIV at this clinic; the present analysis falls within the scope of that protocol, and no amendment was required.

All participants gave written informed consent after receiving information in English or Twi about purpose, procedures, risks, benefits, and the right to withdraw at any time without effect on their care. For participants with limited literacy the information was read aloud and consent recorded by thumbprint witnessed by an impartial observer. Confidentiality was protected through participant codes, password-protected storage, and restriction of access to the research team. No monetary compensation was given; transport costs were reimbursed.

### 2.9 Trustworthiness

Trustworthiness was pursued across credibility, dependability, confirmability, and transferability (35, 36). Credibility was supported by PAB summarising and checking her understanding with participants during interviews. This was in-interview clarification rather than member checking: transcripts were not returned to participants, no repeat interviews were held, and participants did not review the findings.

A second coder with qualitative experience independently coded a subset of transcripts, and discrepancies were resolved by discussion. Peer debriefing with the academic supervisor tested emerging interpretations. Dependability and confirmability rest on an audit trail of coding decisions, category development, and reflexive entries.

Every quotation and participant identifier reported here was verified against the original audio recordings, and code and sub-theme assignments were checked against the utterances they were drawn from. Composite excerpts joining non-contiguous speech were dismantled, so that each excerpt presented below is contiguous speech from a single answer unless an ellipsis marks omission. The verification record, including corrections made to attributions carried from the underlying thesis, is documented in Supplementary Material S1.

Transferability claims are deliberately modest. The account of setting and participants given here remains primarily demographic and institutional, and is offered as sufficient for readers to judge relevance to comparable district clinics rather than as thick description in the ethnographic sense.

## 3 Results

### 3.1 Participant characteristics

Twelve adults took part: nine women and three men. Ages are presented in five-year bands, consistent with the supplementary materials and to reduce re-identification risk given the named single site (Table 1).

Table 1 shows a sample varied across age band, marital status, parity, education, and employment, spanning nulliparous participants and one participant with six children. Partner HIV status is not tabulated because it was not systematically recorded. All participants who had children reported that those children were HIV-negative. This is participants' own report; clinical records were not reviewed, and no verification of children's serostatus was undertaken. The pattern is consistent with what would be expected where prevention of mother-to-child transmission services are received, but this study cannot demonstrate that clinically.

The sample was predominantly female. Nine of twelve participants being women is most plausibly explained by clinic attendance patterns, by the recruitment route through routine appointments, and by willingness to discuss fertility with a female nurse-researcher. The composition of the sample is not treated here as evidence about reproductive expectations, since the study design cannot support such an inference.

### 3.2 Organisation of themes

Four themes are reported (Table 3). The contributing-participant counts are displayed so that the evidential base of each theme is visible, and they should be read as the number of participants whose material was coded to any part of the theme rather than as agreement across those participants.

**Table 3:** Themes and sub-themes reported in this study.

Theme	Sub-themes	Number of participants contributing
Theme 1: Treatment access and service appraisal	Reports of uninterrupted medication access and adequate care (4 participants); a single account linking daily medication-taking to felt bodily wellness (1 participant); a single account of belief that medication is safe in pregnancy (1 participant)	4
Theme 2: Transmission concern and reported prevention information	Fear of infecting a child; provider messages about protecting a baby; one account of a provider message about protecting a partner	6
Theme 3: Reproductive counselling as experienced by patients	Counselling reported at routine visits; one qualified numerical appraisal; one account of counselling not yet received; one account of counselling received but disregarded	5
Theme 4: Heterogeneity of fertility positions	Deferral; limitation after completed family; economic framing; aspiration with uncertainty; partner pressure; persisting anxiety alongside information	8

Table 3 reports what the data support rather than a comprehensive map of reproductive experience. Theme 1 is the narrowest of the four, and its sub-themes are stated with their individual counts because the theme is not internally uniform: four participants spoke about access and service adequacy, but only one linked medication-taking to a felt state of health. Themes 2 and 3 concern prevention information and counselling. Theme 4 is the broadest-based and exists because the dataset's most robust feature is not a single pathway from treatment to positive intention but a spread of reproductive positions that resist that summary.

### 3.3 Theme 1: treatment access and service appraisal

Four participants spoke about access to medication and the adequacy of the care they received (Table 4). The content of these accounts is uneven, and Table 4 is presented with that unevenness visible rather than smoothed.

**Table 4:** Excerpts: treatment access and service appraisal.

Participant	Excerpt	What the excerpt shows
P8 (female, 25–29, single, no children)	“I was very worried from the beginning but as time went by I accepted my reality. I have access and by taking the medications given I feel healthy.”	Links medication-taking to a felt state of wellness, following an earlier period of distress. Does not connect that wellness to childbearing
P9 (female, 45–49, married, three children)	“That even if I want to have a child again, taking my medications will not affect the baby.”	Reports belief, attributed to provider information, that medication is safe in pregnancy. Concerns pharmacological safety, not the participant's own health
P10 (female, 35–39, single, three children)	“I am satisfied with their services.”	Service appraisal. Carries no reproductive content
P7 (male, 45–49, married, six children)	“They give me the needed care.”	Service appraisal. Carries no reproductive content

Table 4 supports a narrow claim. P8's account is the only one in the dataset in which medication-taking is described as producing a felt state of health, and her phrasing places that wellness after a period of worry rather than as an immediate consequence of diagnosis. Even in her case, the account stops short of connecting bodily wellness to a decision about children; the link between feeling well and childbearing is one a reader might infer, but it is not one she states. P9's excerpt addresses a different matter entirely, namely whether medication would harm a baby, which is a belief about pharmacology rather than about her own health, and she did not wish to have further children in any case, as Table 5 shows. The remaining two excerpts in Table 4 are appraisals of service adequacy with no reproductive content at all.

**Table 5:** Excerpts: heterogeneity of fertility positions.

Participant	Excerpt	Position and reasoning
P7 (male, 45–49, married, six children)	“I was not really worried because I have enough kids.”	Diagnosis carried little reproductive weight because childbearing was complete
P7 (male, 45–49, married, six children)	“I don't want children again… I have enough children.” Asked what mattered most in considering children: “Economic factors.”	Limitation framed economically, not around transmission
P7 (male, 45–49, married, six children)	Asked whether HIV status influenced his thinking about children: “No please.”	Explicit divergent case: denies any influence of serostatus on reproductive thinking
P3 (female, 25–29, married, no children)	“I don't have a child yet so I will put this on hold for now… I want to continue my lineage.”	Deferral rather than decision, with lineage as motivation
P6 (female, 35–39, married, one child)	“I am been pressured by my husband and also I have a boy so I want a baby girl too.”	Partner pressure and preference for a child of a particular sex shaping the wish for another child
P6 (female, 35–39, married, one child)	“I want a baby but doesn't want to put him/her at risk because of the disease.”	Wish for a further child held together with transmission concern
P9 (female, 45–49, married, three children)	“Because of the disease I wish to have no more kids but it hasn't affected any area of my life.”	Limitation attributed to HIV, alongside denial of wider life impact
P10 (female, 35–39, single, three children)	“I don't want to complicate the life of any child hence my decision to stick to the ones I have for now.”	Limitation, with “for now” marking provisionality
P8 (female, 25–29, single, no children)	“I am just hopeful that when the time comes for me to have a baby I can also go through my due months and have a healthy baby as any other woman out there.”	Aspiration held with acknowledged uncertainty
P2 (female, 35–39, divorced, one child)	“I wish to have additional children but am scared for the baby though I have been taught that my child can be prevented from getting infected.”	Desire, residual fear, and received information coexisting

The theme is therefore reported as treatment access and service appraisal rather than as an account of perceived health enabling reproductive thinking. Its evidential base for anything beyond access and satisfaction is a single account, and the analysis does not build on it further.

Two additional points bear on how Table 4 should be read. Adherence in these accounts is perceived and self-reported; no participant's adherence was verified, and interviews conducted by a nurse-researcher inside the clinic may have encouraged favourable self-report on both adherence and services. Further, no participant in Table 4, or anywhere in the dataset, referred to a viral load result, to being undetectable, or to viral load monitoring. Treatment appears in these accounts as medication-taking and as service received, not as measured suppression.

### 3.4 Theme 2: transmission concern and reported prevention information

Six participants spoke about fear of infecting a child and about information they had received concerning protection (Table 6).

**Table 6:** Excerpts: transmission concern and reported prevention information.

Participant	Excerpt	What the excerpt shows
P1 (female, 25–29, single, no children)	“I really wish to have children but I am also thinking about whether my children will contract the sickness or not. Also because [of] this I intend not to have children.”	Desire and transmission fear held together, with fear determining the stated position
P10 (female, 35–39, single, three children)	“I was worried that the baby was going to contract the disease but thankfully my baby was not affected.”	Retrospective account of anxiety during a pregnancy that ended with an uninfected child
P8 (female, 25–29, single, no children)	“That having the disease doesn't deter one from not having kids and in our condition we can still have healthy babies.”	Reports the content of provider messaging when asked how providers addressed her concerns
P2 (female, 35–39, divorced, one child)	“There are drugs I can take to [prevent] my baby from having the disease and how I can get pregnant with my partner without infecting him as well.”	Reports counselling content covering both infant and partner protection
P6 (female, 35–39, married, one child)	"That I can still give birth in my condition without affecting my child.”	Reports provider assurance that childbearing is possible without infecting the child
P9 (female, 45–49, married, three children)	“I don't want to infect any child I might have.”	States transmission concern as the reason for not wanting further children

Table 6 requires careful reading. Three of the six excerpts, from P8, P2, and P6, were given in answer to the question asking how healthcare providers addressed their fertility concerns. They are therefore reports of what participants were told, and they establish that certain messages were delivered at this clinic. They do not establish the depth of participants' own understanding.

P2's account in Table 6 is the only statement in the dataset touching on protecting a partner during conception. It is a valuable indication that such counselling occurred, and it is reported here for that reason. It does not demonstrate detailed safer conception knowledge, and it makes no reference to viral suppression.

Table 6 also shows that transmission concern did not operate uniformly. For P1 it was decisive against having children, for P9 it was one reason among others for limiting further births, for P10 it was a remembered anxiety that resolved in a good outcome, and for P6 it was a concern the clinic had addressed with reassurance that childbearing without infecting the child was possible. P6's account is taken up again under Theme 4, because in her case that reassurance sat alongside partner pressure to have a further child.

### 3.5 Theme 3: reproductive counselling as experienced by patients

Five participants described their experience of reproductive counselling at the clinic, and their accounts are not uniform (Table 7).

**Table 7:** Excerpts: reproductive counselling as experienced by patients.

Participant	Excerpt	What the excerpt shows
P2 (female, 35–39, divorced, one child)	“We are being taught every time I visit the hospital and I will rate them about 70%.”	Reports teaching at each visit, with an appraisal short of full satisfaction
P8 (female, 25–29, single, no children)	“Yes please, every time I come for checkup.”	Reports counselling at routine visits
P3 (female, 25–29, married, no children)	“Yes please, we are taught at the hospital after child bearing in our condition and our general wellbeing.”	Reports teaching without specifying frequency; locates it within general wellness education
P1 (female, 25–29, single, no children)	“Not yet, but I think along the line I will receive counselling on fertility issues.”	Reports fertility-specific counselling not yet received
P7 (male, 45–49, married, six children)	“Yes but I don't pay much attention because I have enough kids.”	Counselling received but not attended to, given a settled reproductive position

Table 7 supports the statement that four of these five participants reported receiving reproductive teaching or counselling, two of them describing it as occurring at every visit, and that one reported not having received fertility-specific counselling. It does not support a claim of systematic, clinic-wide integration of reproductive health into HIV care, which would require provider interviews, observation, or service records that this study did not collect.

Two accounts in Table 7 diverge from a straightforward routine-provision reading and are retained deliberately. P1 reports that she had not yet received fertility-specific counselling, which cuts against the accounts of teaching at every visit. P7 reports receiving counselling and disregarding it, because his reproductive position was already settled. Together these suggest that counselling neither reached every participant nor landed equally where it did, and that its relevance is filtered by where a patient stands.

P2’s numerical appraisal in Table 7 is a single participant’s rating, given in response to a general question about her experience of health services rather than to any scaled instrument. What she meant by 70% was not probed, and no other participant offered a numerical rating. It is reported as one participant’s qualified appraisal and nothing more, and no pattern is drawn from it.

### 3.6 Theme 4: heterogeneity of fertility positions, including men’s accounts

The dataset’s most consistent feature is the diversity of reproductive positions and the reasoning behind them (Table 5). This theme also carries the study’s one substantive male account and the single account in which a partner is named as shaping a childbearing decision.

Table 5 shows that participants occupied at least four distinguishable positions: deferral, aspiration held with uncertainty, limitation after a completed family, and settled non-intention. Framing these uniformly as positive fertility intentions supported by treatment would misrepresent the dataset.

P7’s account, presented across three rows of Table 5, is the study's clearest divergent case. Asked directly whether his HIV status influenced his thinking about children, he answered that it did not. He identified economic factors as paramount, described himself as unworried at diagnosis because his family was complete, and reported that he paid little attention to fertility counselling for the same reason. His account contains no reference to transmission fear, to prevention of mother-to-child transmission, or to treatment as a condition of parenthood. Taken together with P9, who also reported completed childbearing, Table 5 suggests that parity may organise reproductive thinking at this clinic at least as strongly as HIV status does, a possibility this study can raise but not test.

P6's account is the only one in which a partner is named as exerting influence on a childbearing decision, and it is reported because it speaks to how relational dynamics can enter reproductive thinking. She describes pressure from her husband, combined with a preference for a child of a particular sex, as the reason for wanting another child, while in the same interview holding a clear concern about not putting that child at risk. Her account is a single instance and cannot describe partner involvement across the sample; the study did not interview partners, and the guide did not ask systematically about how decisions were negotiated within couples. It does indicate that for at least one participant the wish for a further child was not formed in isolation.

Substantive fertility-related content was available from one man only. The material for the two other male participants did not speak substantively to the reported themes, and male representation has not been extended by drawing on thin material. This constrains what the study can say about gendered reproductive decision-making, and it means the findings should not be read as speaking equally for women and men.

Table 2 makes the evidential base of each theme explicit. Eight of twelve participants contributed to Theme 4, which is the broadest-based finding. Theme 1 rests on four accounts and, as Section 3.3 sets out, on a single account for anything beyond access and service appraisal. Four participants (P4, P5, P11, P12) do not appear in the excerpt tables, because the material available for them did not speak substantively to the reported themes. Presenting this distribution openly is preferable to language implying whole-sample agreement.

### 3.7 Schematic of themes

Figure 1 presents the four themes as co-present influences on the reproductive positions participants described. It was developed from the completed analysis rather than specified in advance, and it deliberately carries no directional arrows, since the study establishes neither ordering nor causation among the themes.

**Figure 1:**
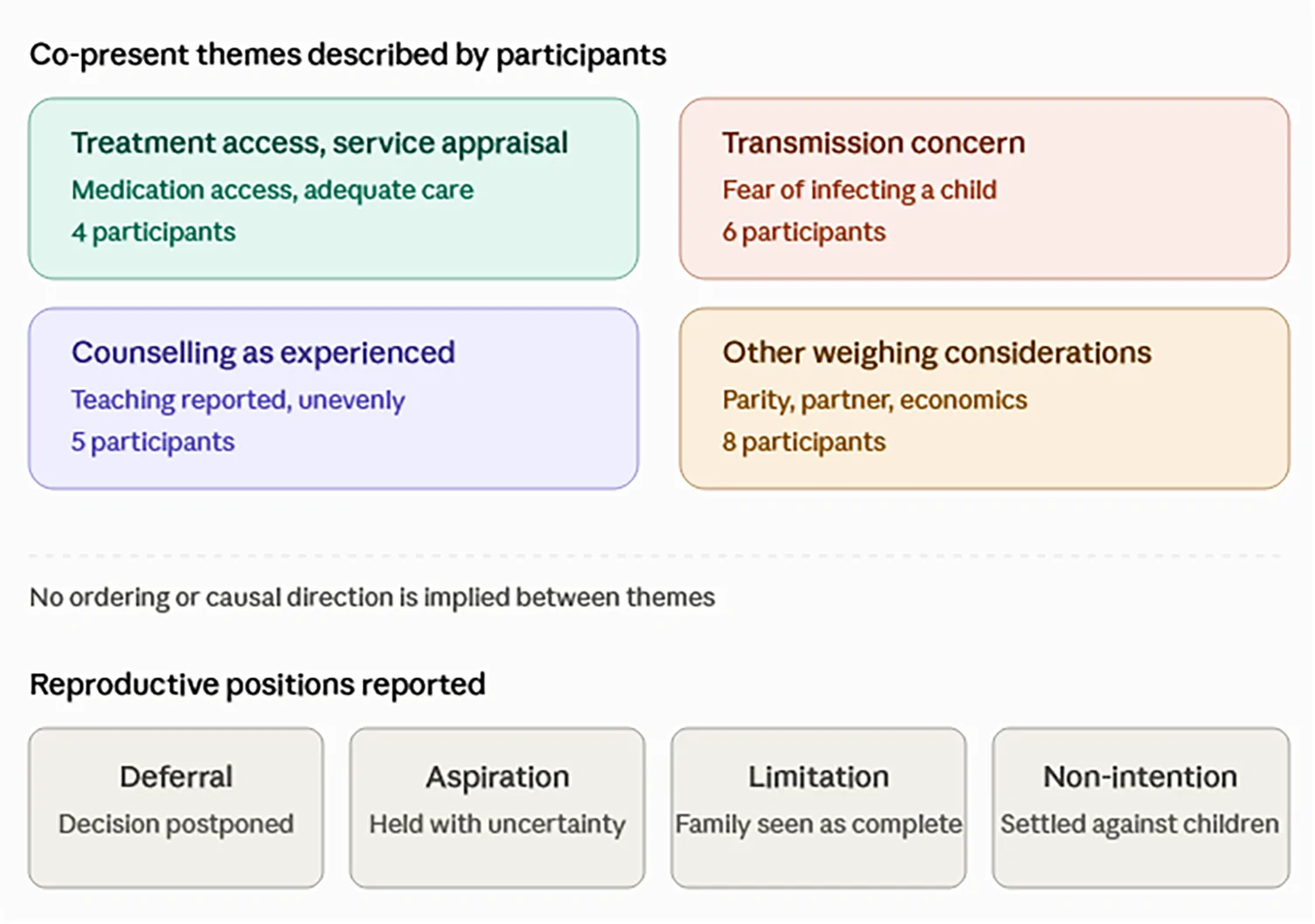
Descriptive schematic of themes contributing to reproductive positions among participants at one district antiretroviral therapy clinic, Ashanti Region, Ghana.

## 4 Discussion

This study asked how people living with HIV at one district antiretroviral therapy clinic in Ghana described the place of treatment in their reproductive thinking. Three claims are supported. Treatment access and clinic services were described as adequate by several participants, though the accounts rarely extended from treatment to reproductive thinking. Prevention information reached patients, primarily as messages about protecting a baby. Reproductive positions were heterogeneous and were organised by parity, partnership, economics, and emotion at least as much as by HIV status. A fourth claim, that viral suppression shaped these decisions, is not supported and is not advanced.

### 4.1 What these data can and cannot say about viral suppression

No participant referred to a viral load result, to undetectable status, or to viral load monitoring, and the interview guide did not ask about these. Participants' talk concerned taking medication, receiving care, and being told that a baby could be protected. Analytically, these are distinct: perceived adherence is not laboratory-confirmed suppression, knowledge that medication protects an infant is not knowledge of viral suppression, and neither is equivalent to understanding treatment as prevention.

This distinction matters because the surrounding evidence base is strong and could easily be read back into the data. The PARTNER studies and HPTN 052 established that sustained suppression prevents sexual transmission, with HPTN 052 reporting a 93% reduction in linked transmission (9–12). Ghanaian data show that durable suppression is achievable locally, with 88% of one cohort below 50 copies per millilitre at eighteen months (8), even as roughly a quarter of patients in a larger western-region sample remained unsuppressed (7) and national adherence sits below the level suppression requires (6). All of this is clinically important. None of it can be inferred from what these twelve participants said.

Treatment as prevention is therefore presented here as a service implication rather than a finding. If patients at district clinics receive prevention information framed almost entirely around infant protection, as these accounts suggest, then the partner-protective dimension of suppression may not be reaching them in a usable form. That would be consistent with regional evidence that safer conception counselling is uneven and often centres the woman and child rather than the couple (20, 21). Establishing whether this is so at this clinic would require research designed for the purpose, including questions about viral load understanding and interviews with providers.

### 4.2 Treatment access, perceived health, and the limits of what these accounts show

The relationship between treatment and reproductive thinking is where this study's data are thinnest, and the finding is best stated as a negative one. Four participants described access and services positively, but only one linked medication-taking to a felt state of wellness, and no participant explicitly connected that wellness to a decision about children.

This matters because the regional literature would predict otherwise. In Soweto, among 442 patients on treatment, feeling healthy after starting therapy was among the reasons participants gave for desiring children, and 47% expressed such a desire (15). Broader work has documented sustained fertility aspiration accompanying treatment scale-up (13, 14), and qualitative research in western Kenya found that treatment influenced how women appraised the possibility of childbearing (37). The present accounts do not reproduce that pattern with any strength. One reading is that the link exists at this clinic but was not elicited, since the guide asked about health and about children separately rather than about the relationship between them. Another is that treatment had become sufficiently routine for these participants that it functioned as background rather than as something they weighed when thinking about children, which would be consistent with P7's flat denial that his serostatus bore on the question at all. The study cannot distinguish these, and the honest position is that a link frequently reported elsewhere is not demonstrable here.

The Theory of Planned Behaviour offers one available reading of the single account that does connect medication and wellness, in which belief that treatment secures health functions as perceived behavioural control supporting intention (25, 26). We offer this interpretively and note its limits plainly: the theory did not shape data collection or coding, one account cannot test a model, and P7's case sits outside its explanatory reach entirely.

The practical implication is correspondingly modest. Adherence support remains clinically essential, particularly given that adherence in Ghana falls below the threshold suppression requires and is lowest among younger patients (6). Whether it also carries reproductive significance is a proposition these data raise without settling.

### 4.3 Heterogeneity, parity, and the limits of a gendered reading

The spread of reproductive positions in these accounts is their most robust feature. Two participants with completed families, P7 and P9, described HIV as largely irrelevant or secondary to their reproductive positions, while participants without children, P1, P3, and P8, described deferral, aspiration, and fear. Parity emerges as an organising consideration, consistent with quantitative findings that absence of a biological child is strongly associated with fertility desire (AOR 5.06, 95% CI 2.11 to 12.1) (15) and with regional evidence linking lower parity and partner desire to childbearing intention (38, 39).

Relational influence appears directly in only one account, that of P6, for whom pressure from a husband and a preference for a child of a particular sex shaped the wish for another child. A single instance cannot establish how couples negotiate these decisions, but it is consistent with regional evidence that a partner's desire for children is among the more consistent correlates of fertility intention among people living with HIV (39), and it reinforces the case for couple-based inquiry.

Gender is where this study is weakest. Only one man's account contributed substantively, and it is a distinctive one: economic framing, completed family, and explicit denial that serostatus mattered. It would be unsound to generalise from it. The wider literature indicates that men's reproductive desires are often stronger than women's, with adjusted odds of desiring a child more than four times higher among men in one Nairobi study (OR 4.17, 95% CI 2.46 to 7.07) (16), and that men frequently drive conception decisions while rarely receiving safer conception advice (20, 37).

The thinness of male material here is itself worth noting, because it echoes a structural pattern rather than being merely a sampling accident. Men are consistently underrepresented across the HIV care continuum in sub-Saharan Africa, and a scoping review of 17 qualitative studies across 8 countries identified masculine norms, including the perception that clinics are spaces for women, as barriers to men's engagement in care (40). A clinic-based recruitment route in a facility whose reproductive services are oriented toward women will tend to reproduce that pattern in a research sample. Future work at this setting should sample men purposively, recruit outside routine appointment attendance, and interview partners or couples.

Relatedly, the study's ability to speak about decision-making as such is limited. The guide asked about thoughts, reasons, and influences rather than about how and when decisions were reached, whether partners participated, or how contraception and spacing featured. Several accounts describe positions rather than decisions, and P10's “for now” and P3's “on hold” suggest provisionality that a cross-sectional interview cannot track. Work elsewhere has shown that fertility intentions among women living with HIV shift over time (41), which argues for longitudinal designs.

### 4.4 Counselling, the institutional setting, and religious context

Four participants reported receiving reproductive counselling or teaching, two of them describing it as occurring at every visit, and one reported not having received fertility-specific counselling. This supports the observation that teaching occurs and that it does not reach everyone; it does not support conclusions about coverage, consistency, or quality, which would require provider and service-level data. Counselling for women living with HIV in low and middle income settings does improve knowledge and service use where it is delivered well (22), and integration of reproductive services into HIV care remains widely recommended (18, 19). Whether this clinic achieves that is a question our design cannot answer.

The institutional context deserves explicit attention. The study site is a Catholic mission hospital within the Christian Health Association of Ghana network. Catholic facilities constitute the largest denominational bloc in that network and do not provide artificial contraception, with clients requesting such methods referred to other facilities; training in methods such as implant insertion excludes Catholic institutions, and natural methods are the ones supported in-house (31). This has plausible implications for the reproductive counselling participants received. Counselling oriented toward achieving safe pregnancy might be delivered readily in such a setting, while counselling on limiting or spacing births through modern contraception might be delivered differently, elsewhere, or not at all. That asymmetry could help explain why the accounts in this study contain considerably more material about protecting a baby than about preventing or spacing pregnancy, notwithstanding that several participants wished to have no further children.

This is advanced as a hypothesis for investigation, not a finding. Participants were not asked about contraception, about religious influence on their care, or about referral elsewhere, and the study collected no data on what the clinic does or does not provide. Participants' own denominations, where mentioned, were not Catholic, so the relevant influence would be institutional rather than personal. Research examining how faith-based facilities in Ghana counsel people living with HIV on the full range of reproductive options, including limitation as well as conception, would be valuable.

### 4.5 Emotion alongside information

Several participants held accurate prevention information and remained anxious. P2 described being scared for a baby despite having been taught that infection could be prevented, P6 described wanting a baby while not wanting to put the child at risk, and P1 described a wish for children negated by fear of transmission. This pattern is well documented in reproductive research with people living with HIV, where transmission fears persist alongside effective interventions (42, 43).

An important qualification applies. Because no participant's viral load was verified or discussed, we cannot say whether anxiety persisted despite confirmed suppression, despite perceived adherence, or despite general prevention information. These are different situations with different clinical implications, and this study cannot distinguish them. What it can say is that information and reassurance were not equivalent for these participants, which supports counselling that addresses emotional response alongside biomedical content.

### 4.6 Strengths and limitations

The study offers patient-centred accounts from a district-level West African setting that is underrepresented in this literature, verifies every quotation against source recordings, and reports divergent cases alongside patterned ones.

Its limitations are substantial and are stated plainly. It is a single-site study of twelve participants and does not describe people living with HIV in Ghana generally. The material available for four participants did not speak substantively to any reported theme, so the effective analytic sample was eight, and saturation is not claimed. Substantive fertility content was available from one man only, so the study cannot speak to gendered decision-making. The link between treatment, perceived health, and reproductive thinking rests on a single account and is reported as undemonstrated rather than as established. Viral suppression was neither verified nor elicited. Adherence and service appraisals were reported to a nurse-researcher inside the clinic, so favourable reporting is plausible. Partner HIV status was not systematically recorded, and partner involvement in decisions is evidenced by one account. Fertility decision-making processes, contraception, and spacing were not directly explored. Transcription and translation from Twi were performed in a single step by one bilingual researcher without independent back-translation. The interview guide was not formally pilot tested. Recruitment counts were not documented. Transcripts were not returned to participants and findings were not member-checked.

## 5 Conclusion

Among twelve adults attending one district antiretroviral therapy clinic in Ghana's Ashanti Region, treatment access and clinic services were described as adequate, and prevention information reached patients mainly in the form of assurance that a baby could be protected. The connection between treatment, feeling well, and thinking about children, which regional studies frequently report, appeared in only one account here and was not stated by any participant as a reason for a reproductive decision. Reproductive positions were not uniform: participants deferred, aspired with uncertainty, limited births after completing their families, or reported that HIV bore no relation to their reproductive thinking at all, with parity, partnership, economics, and emotion all shaping where they stood.

These data do not establish that viral suppression drives fertility decision-making. No participant discussed viral load, and none was clinically verified as suppressed. Nor do they establish that perceived health enabled positive reproductive intentions, since that link is evidenced by a single account. The defensible reading is that reproductive positions at this clinic were shaped by a range of considerations in which HIV status carried variable and sometimes minimal weight. Because men's accounts are minimally represented, the findings should not be read as speaking equally for women and men.

For services in comparable settings, the implications are that prevention counselling framed around infant protection may leave the partner-protective dimension of treatment underexplored, that counselling did not reach every participant and did not land equally where it did, and that accurate information alone did not resolve participants’ emotional uncertainty. Research designed to examine viral load understanding directly, to recruit men beyond routine clinic attendance, to include partners, and to examine how faith-based facilities counsel across the full range of reproductive options would build usefully on this account.

## Data Availability

The qualitative datasets analysed in this study contain potentially identifying information and are therefore not publicly available. De-identified excerpts supporting the findings of this study are included in the article/Supplementary Material. Further de-identified data may be made available by the corresponding author on reasonable request, subject to ethics approval.

## Supplementary material

SUPPLEMENTARY MATERIAL S1 De-identified qualitative excerpts and coded thematic summaries.

SUPPLEMENTARY MATERIAL S2 Completed COREQ 32-item checklist.
