# Supplementary material for: Antiretroviral therapy, perceived health, and fertility decision-making among people living with HIV: a qualitative study at a district antiretroviral therapy clinic in the Ashanti Region, Ghana
Source: Front Reprod Health. 2026 Sep 29;8:1914707. doi: 10.3389/frph.2026.1914707 (PMC13658555; doi:10.3389/frph.2026.1914707)
Supplement: Supplementary file 1 [file Supplementaryfile1.docx]

**Supplementary Material S1**

**De-identified qualitative excerpts and coded thematic summaries**

**Manuscript:** Antiretroviral therapy, perceived health, and fertility decision-making among people living with HIV: a qualitative study at a district antiretroviral therapy clinic in the Ashanti Region, Ghana

**Authors:** Priscilla Asantewaa Boadi, Victor Luckyboy Dzramado

**Journal:** Frontiers in Reproductive Health. Manuscript ID 1914707. doi: 10.3389/frph.2026.1914707

## **Note on de-identification and conventions**

All participants are adults living with HIV who were receiving antiretroviral therapy at the study clinic. Each participant is identified only by an anonymised code (P1 to P12). No names, facility unit identifiers, or other directly identifying details appear. Ages are reported in five-year bands rather than exact ages to strengthen confidentiality, consistent with the article.

Each excerpt reproduced below is contiguous speech from a single answer unless an ellipsis marks omission. Square brackets mark an editorial insertion for readability. Grammar and syntax are left unaltered, so non-standard English constructions reflect either the participant’s own English or the researcher’s close rendering of Twi speech. Composite excerpts joining non-contiguous speech, which appeared in an earlier version of this file, have been dismantled; the corrections are recorded in Part 5.

This file documents the coding framework and the de-identified excerpts underlying the four themes reported in the article. The study did not ask about, verify, or elicit viral load results, undetectable status, or viral load monitoring. No excerpt in this file should be read as evidence about viral suppression.

# **Part 1. Codebook and coding framework**

**AUTHOR ACTION REQUIRED.** Codes C15 to C23 have been reconstructed from the interview transcripts to complete the codebook to the twenty-three inductive codes reported in Section 2.7 of the article and in item 25 of the COREQ checklist. They are grounded in participants’ own language, and every excerpt in Part 2 now carries an assignment. They are not necessarily identical to the labels used in the original coding register. Please check C15 to C23 against that register and amend any label or definition that differs before this file is uploaded.

**AUTHOR ACTION REQUIRED.** Code C13 was previously labelled “Perceived behavioural control”. The article states that the Theory of Planned Behaviour did not inform the coding frame and was introduced only at the interpretation stage, so a TPB construct cannot appear as an inductive code label. The label below has been restated in participants’ own terms. Please confirm the wording.

**Table S1. Codebook of twenty-three inductive codes.**

| **Code** | **Code label** | **Working definition** | **Illustrative participant language** | **Theme** |
| --- | --- | --- | --- | --- |
| C1 | Access to medication and services | Reliable, free access to antiretroviral therapy and clinic services | “I have access…”; “I receive all the necessary services” | 1 |
| C2 | Medication-taking and felt health | Daily medication-taking described as producing a sense of being healthy | “by taking the medications given I feel healthy” | 1 |
| C3 | Medication safety in pregnancy | Belief that treatment does not harm a future baby | “taking my medications will not affect the baby” | 1 |
| C4 | Reported assurance about protecting a baby | Provider message that transmission to a child can be prevented | “I can still give birth in my condition without affecting my child” | 2 |
| C5 | Reported information on protecting a partner | Provider message about conceiving without infecting a partner | “how I can get pregnant with my partner without infecting him as well” | 2 |
| C6 | Fear of infecting a child | Fear of passing HIV to a child, current or retrospective | “I don’t want to infect any child I might have” | 2 |
| C7 | Persisting anxiety alongside information | Worry about reproductive outcomes held together with accurate information | “am scared for the baby though I have been taught that my child can be prevented” | 2, 4 |
| C8 | Normalisation of childbearing | Childbearing described as possible in the same terms as for anyone else | “in our condition we can still have healthy babies” | 2 |
| C9 | Counselling reported at routine visits | Reproductive teaching or counselling described as occurring at clinic visits | “every time I come for checkup” | 3 |
| C10 | Qualified appraisal of counselling | Counselling rated short of full satisfaction | “I will rate them about 70%” | 3 |
| C11 | Counselling not yet received | Fertility-specific counselling reported as not yet given | “Not yet, but I think along the line I will receive counselling” | 3 |
| C12 | Absence of a partner as a constraint | No current partner named as bearing on childbearing | “I don’t have a partner at the moment” | 4 |
| C13 | Sense of capacity for safe childbearing | Stated sense that safe childbearing is achievable for oneself | “I can also go through my due months and have a healthy baby” | 4 |
| C14 | Health placed above other considerations | Health named as the leading factor in the position held | “Health” named first among factors that matter most | 1, 4 |
| C15 | Service appraisal without reproductive content | Satisfaction with care expressed with no reference to childbearing | “I am satisfied with their services”; “They give me the needed care” | 1 |
| C16 | Counselling received but disregarded | Counselling acknowledged but not attended to, given a settled position | “I don’t pay much attention because I have enough kids” | 3 |
| C17 | Deferral of a childbearing decision | Decision postponed rather than made, often marked “for now” | “I will put this on hold for now” | 4 |
| C18 | Lineage and family continuity | Continuing the family line given as a reason for wanting a child | “I want to continue my lineage” | 4 |
| C19 | Completed family as reason for limitation | Existing children given as the reason for wanting no more | “I have enough children”; “I am content with the 3 kids” | 4 |
| C20 | Economic considerations | Financial capacity named as bearing on the decision | “Economic factors” | 4 |
| C21 | Partner pressure and child-sex preference | A partner’s wish, or a preference for a child of a particular sex, shaping the position | “I am been pressured by my husband and also I have a boy so I want a baby girl too” | 4 |
| C22 | Settled non-intention | A decided position against having children | “I intend not to have children”; “I don’t want children again” | 4 |
| C23 | Serostatus denied as an influence | HIV status explicitly stated not to bear on thinking about children | Asked whether HIV status influenced his thinking: “No please” | 4 |

**Table S2. Mapping of codes to sub-themes and to the themes reported in the article.**

| **Theme** | **Sub-theme** | **Contributing codes** |
| --- | --- | --- |
| Theme 1: Treatment access and service appraisal | Reports of uninterrupted medication access and adequate care (4 participants) | C1 |
| Theme 1: Treatment access and service appraisal | A single account linking daily medication-taking to felt bodily wellness (1 participant) | C2, C14 |
| Theme 1: Treatment access and service appraisal | A single account of belief that medication is safe in pregnancy (1 participant) | C3 |
| Theme 2: Transmission concern and reported prevention information | Fear of infecting a child | C6 |
| Theme 2: Transmission concern and reported prevention information | Provider messages about protecting a baby | C4, C8 |
| Theme 2: Transmission concern and reported prevention information | One account of a provider message about protecting a partner | C5 |
| Theme 3: Reproductive counselling as experienced by patients | Counselling reported at routine visits | C9 |
| Theme 3: Reproductive counselling as experienced by patients | One qualified numerical appraisal | C10 |
| Theme 3: Reproductive counselling as experienced by patients | One account of counselling not yet received | C11 |
| Theme 3: Reproductive counselling as experienced by patients | One account of counselling received but disregarded | C16 |
| Theme 4: Heterogeneity of fertility positions | Deferral | C17, C18 |
| Theme 4: Heterogeneity of fertility positions | Limitation after a completed family | C19, C14 |
| Theme 4: Heterogeneity of fertility positions | Economic framing | C20 |
| Theme 4: Heterogeneity of fertility positions | Aspiration held with uncertainty | C13, C8 |
| Theme 4: Heterogeneity of fertility positions | Partner pressure | C21, C12 |
| Theme 4: Heterogeneity of fertility positions | Persisting anxiety alongside information | C7, C6 |
| Theme 4: Heterogeneity of fertility positions | Settled non-intention, including the divergent male case | C22, C23 |

# **Part 2. De-identified excerpts by theme**

The excerpts and attributions below are those verified against the original audio recordings and reported in the article. They correspond to Tables 4 to 7 of the published article.

## **Theme 1. Treatment access and service appraisal**

**Table S3. Excerpts: treatment access and service appraisal.**

| **Participant** | **De-identified verbatim excerpt** | **What the excerpt shows** | **Codes** |
| --- | --- | --- | --- |
| P8 (female, 25–29, single, no children) | “I was very worried from the beginning but as time went by I accepted my reality. I have access and by taking the medications given I feel healthy.” | Links medication-taking to a felt state of wellness, following an earlier period of distress. Does not connect that wellness to childbearing | C1, C2, C14 |
| P9 (female, 45–49, married, three children) | “That even if I want to have a child again, taking my medications will not affect the baby.” | Reports belief, attributed to provider information, that medication is safe in pregnancy. Concerns pharmacological safety, not the participant’s own health | C3 |
| P10 (female, 35–39, single, three children) | “I am satisfied with their services.” | Service appraisal. Carries no reproductive content | C15 |
| P7 (male, 45–49, married, six children) | “They give me the needed care.” | Service appraisal. Carries no reproductive content | C15 |

## **Theme 2. Transmission concern and reported prevention information**

**Table S4. Excerpts: transmission concern and reported prevention information.**

| **Participant** | **De-identified verbatim excerpt** | **What the excerpt shows** | **Codes** |
| --- | --- | --- | --- |
| P1 (female, 25–29, single, no children) | “I really wish to have children but I am also thinking about whether my children will contract the sickness or not. Also because [of] this I intend not to have children.” | Desire and transmission fear held together, with fear determining the stated position | C6, C7, C22 |
| P10 (female, 35–39, single, three children) | “I was worried that the baby was going to contract the disease but thankfully my baby was not affected.” | Retrospective account of anxiety during a pregnancy that ended with an uninfected child | C6, C7 |
| P8 (female, 25–29, single, no children) | “That having the disease doesn’t deter one from not having kids and in our condition we can still have healthy babies.” | Reports the content of provider messaging when asked how providers addressed her concerns | C4, C8 |
| P2 (female, 35–39, divorced, one child) | “There are drugs I can take to [prevent] my baby from having the disease and how I can get pregnant with my partner without infecting him as well.” | Reports counselling content covering both infant and partner protection | C4, C5 |
| P6 (female, 35–39, married, one child) | “That I can still give birth in my condition without affecting my child.” | Reports provider assurance that childbearing is possible without infecting the child | C4, C8 |
| P9 (female, 45–49, married, three children) | “I don’t want to infect any child I might have.” | States transmission concern as the reason for not wanting further children | C6, C19 |

## **Theme 3. Reproductive counselling as experienced by patients**

**Table S5. Excerpts: reproductive counselling as experienced by patients.**

| **Participant** | **De-identified verbatim excerpt** | **What the excerpt shows** | **Codes** |
| --- | --- | --- | --- |
| P2 (female, 35–39, divorced, one child) | “We are being taught every time I visit the hospital and I will rate them about 70%.” | Reports teaching at each visit, with an appraisal short of full satisfaction | C9, C10 |
| P8 (female, 25–29, single, no children) | “Yes please, every time I come for checkup.” | Reports counselling at routine visits | C9 |
| P3 (female, 25–29, married, no children) | “Yes please, we are taught at the hospital after child bearing in our condition and our general wellbeing.” | Reports teaching without specifying frequency; locates it within general wellness education | C9 |
| P1 (female, 25–29, single, no children) | “Not yet, but I think along the line I will receive counselling on fertility issues.” | Reports fertility-specific counselling not yet received | C11 |
| P7 (male, 45–49, married, six children) | “Yes but I don’t pay much attention because I have enough kids.” | Counselling received but not attended to, given a settled reproductive position | C16, C19 |

## **Theme 4. Heterogeneity of fertility positions**

**Table S6. Excerpts: heterogeneity of fertility positions.**

| **Participant** | **De-identified verbatim excerpt** | **Position and reasoning** | **Codes** |
| --- | --- | --- | --- |
| P7 (male, 45–49, married, six children) | “I was not really worried because I have enough kids.” | Diagnosis carried little reproductive weight because childbearing was complete | C19 |
| P7 (male, 45–49, married, six children) | “I don’t want children again… I have enough children.” Asked what mattered most in considering children: “Economic factors.” | Limitation framed economically, not around transmission | C19, C20, C22 |
| P7 (male, 45–49, married, six children) | Asked whether HIV status influenced his thinking about children: “No please.” | Explicit divergent case: denies any influence of serostatus on reproductive thinking | C23 |
| P3 (female, 25–29, married, no children) | “I don’t have a child yet so I will put this on hold for now… I want to continue my lineage.” | Deferral rather than decision, with lineage as motivation | C17, C18 |
| P6 (female, 35–39, married, one child) | “I am been pressured by my husband and also I have a boy so I want a baby girl too.” | Partner pressure and preference for a child of a particular sex shaping the wish for another child | C21 |
| P6 (female, 35–39, married, one child) | “I want a baby but doesn’t want to put him/her at risk because of the disease.” | Wish for a further child held together with transmission concern | C6, C7, C13 |
| P9 (female, 45–49, married, three children) | “Because of the disease I wish to have no more kids but it hasn’t affected any area of my life.” | Limitation attributed to HIV, alongside denial of wider life impact | C6, C19, C14 |
| P10 (female, 35–39, single, three children) | “I don’t want to complicate the life of any child hence my decision to stick to the ones I have for now.” | Limitation, with “for now” marking provisionality | C17, C19 |
| P8 (female, 25–29, single, no children) | “I am just hopeful that when the time comes for me to have a baby I can also go through my due months and have a healthy baby as any other woman out there.” | Aspiration held with acknowledged uncertainty | C8, C13, C7 |
| P2 (female, 35–39, divorced, one child) | “I wish to have additional children but am scared for the baby though I have been taught that my child can be prevented from getting infected.” | Desire, residual fear, and received information coexisting | C4, C7, C12 |

# **Part 3. Participant contribution by theme**

**Table S7. Participant contribution by theme.**

| **Theme** | **Contributing participants** | **Number** |
| --- | --- | --- |
| Theme 1: Treatment access and service appraisal | P7, P8, P9, P10 | 4 |
| Theme 2: Transmission concern and reported prevention information | P1, P2, P6, P8, P9, P10 | 6 |
| Theme 3: Reproductive counselling as experienced by patients | P1, P2, P3, P7, P8 | 5 |
| Theme 4: Heterogeneity of fertility positions | P1, P2, P3, P6, P7, P8, P9, P10 | 8 |

Counts are the number of participants whose material was coded to any part of the theme, not agreement across those participants. Four participants (P4, P5, P11, P12) do not appear in the excerpt tables because the material available for them did not speak substantively to the reported themes.

# **Part 4. Coded thematic summaries**

**Theme 1. Treatment access and service appraisal.**

Four participants spoke about access to medication and the adequacy of the care they received. The content of these accounts is uneven and is presented with that unevenness visible. P8’s account is the only one in the dataset in which medication-taking is described as producing a felt state of health, and her phrasing places that wellness after a period of worry rather than as an immediate consequence of diagnosis. Even in her case the account stops short of connecting bodily wellness to a decision about children. P9’s excerpt addresses whether medication would harm a baby, which is a belief about pharmacology rather than about her own health, and she did not wish to have further children in any case. The remaining two excerpts are appraisals of service adequacy with no reproductive content. Adherence in these accounts is perceived and self-reported; no participant’s adherence was verified, and no participant here or anywhere in the dataset referred to a viral load result, to being undetectable, or to viral load monitoring.

**Theme 2. Transmission concern and reported prevention information.**

Six participants spoke about fear of infecting a child and about information they had received concerning protection. Three of the six excerpts, from P8, P2 and P6, were given in answer to the question asking how healthcare providers addressed their fertility concerns. They are therefore reports of what participants were told, and they establish that certain messages were delivered at this clinic. They do not establish the depth of participants’ own understanding. P2’s account is the only statement in the dataset touching on protecting a partner during conception; it is a valuable indication that such counselling occurred, but it does not demonstrate detailed safer conception knowledge and makes no reference to viral suppression. Transmission concern did not operate uniformly: for P1 it was decisive against having children, for P9 it was one reason among others for limiting further births, for P10 it was a remembered anxiety that resolved in a good outcome, and for P6 it was a concern the clinic had addressed with reassurance.

**Theme 3. Reproductive counselling as experienced by patients.**

Five participants described their experience of reproductive counselling at the clinic, and their accounts are not uniform. Four of the five reported receiving reproductive teaching or counselling, two of them describing it as occurring at every visit, and one reported not having received fertility-specific counselling. This does not support a claim of systematic, clinic-wide integration of reproductive health into HIV care, which would require provider interviews, observation or service records that this study did not collect. Two accounts diverge from a straightforward routine-provision reading and are retained deliberately. P1 reports that she had not yet received fertility-specific counselling, which cuts against the accounts of teaching at every visit. P7 reports receiving counselling and disregarding it, because his reproductive position was already settled. P2’s numerical appraisal is a single participant’s rating, given in response to a general question about her experience of health services rather than to any scaled instrument. What she meant by 70% was not probed, no other participant offered a numerical rating, and no pattern is drawn from it.

**Theme 4. Heterogeneity of fertility positions.**

Eight of twelve participants contributed to this theme, which is the broadest-based finding. Participants occupied at least four distinguishable positions: deferral, aspiration held with uncertainty, limitation after a completed family, and settled non-intention. P7’s account, presented across three rows of Table S6, is the study’s clearest divergent case. Asked directly whether his HIV status influenced his thinking about children, he answered that it did not; he identified economic factors as paramount, described himself as unworried at diagnosis because his family was complete, and reported paying little attention to fertility counselling for the same reason. P6’s account is the only one in which a partner is named as exerting influence on a childbearing decision. Substantive fertility-related content was available from one man only, so this material does not speak equally for women and men.

## **Note on interpretation**

This file documents what the coded material contains. It does not support a reading in which adherence, or the viral suppression that adherence secures, enables positive fertility decision-making. No participant referred to a viral load result, to undetectable status, or to viral load monitoring, and the interview guide did not ask about these. The link between treatment, perceived health and reproductive thinking rests on a single account and is reported in the article as undemonstrated rather than established. The Theory of Planned Behaviour did not inform the interview guide or the coding frame; it was introduced only at the interpretation stage, and its application is confined to the Discussion.

# **Part 5. Verification record**

Section 2.9 of the article states that every quotation and participant identifier was verified against the original audio recordings, that composite excerpts joining non-contiguous speech were dismantled, and that the corrections made to attributions carried from the underlying thesis are documented in this file. The table below records those corrections.

**AUTHOR ACTION REQUIRED.** This verification record has been reconstructed by comparing the superseded version of this supplementary file against the verified excerpt tables in the accepted article. Please check each row against your own verification notes and confirm or amend before this file is uploaded. Row 7 in particular records an excerpt that does not appear in the published tables; the reason for its withdrawal needs to be stated by the authors.

**Table S8. Corrections to excerpts and attributions carried from the underlying thesis.**

| **#** | **Excerpt as carried from the thesis** | **Attributed to** | **Verified attribution** | **Nature of correction** |
| --- | --- | --- | --- | --- |
| 1 | “Every time I come for checkup. We are being taught every time I visit the hospital and I will rate them about 70%.” | P6 | “Yes please, every time I come for checkup.” → P8. “We are being taught every time I visit… about 70%.” → P2 | Composite of two participants’ speech. Dismantled into two excerpts; both attributions corrected |
| 2 | “We are being taught every time I visit the hospital and I will rate them about 70%.” | P6 | P2 | Attribution corrected |
| 3 | “There are drugs I can take to [prevent] my baby from having the disease and how I can get pregnant with my partner without infecting him as well.” | P6 | P2 | Attribution corrected. This is the dataset’s only account of partner protection, so the correction is material |
| 4 | “I am satisfied with their services. That I can still give birth any time and have a healthy baby.” | P10 | “I am satisfied with their services.” → P10. “That I can still give birth in my condition without affecting my child.” → P6 | Composite dismantled; second clause reattributed and restored to its recorded wording |
| 5 | “They give me the needed care. That even if I want to have a child again, taking my medications will not affect the baby.” | P9 | “They give me the needed care.” → P7. “That even if I want to have a child again…” → P9 | Composite dismantled; first clause reattributed |
| 6 | “That I can still give birth any time and have a healthy baby. We are being taught…” | P10 | “That I can still give birth in my condition without affecting my child.” → P6 | Composite dismantled; reattributed and restored to its recorded wording |
| 7 | “I was told that you will be put on medications to protect the unborn baby.” | P3 | Not retained in the published excerpt tables | Traced to a different transcript (the second audio-recorded interview, a participant whose material was not among the eight contributing accounts), not to P3. Withdrawn rather than reattributed. [Authors to confirm the participant code] |
| 8 | No excerpt from any male participant | — | P7 (male, 45–49) contributes three excerpts to Theme 4 and one each to Themes 1 and 3 | The study’s one substantive male account, and its clearest divergent case, was absent from the earlier file and has been restored |
