# Supplementary material for: Antiretroviral therapy, perceived health, and fertility decision-making among people living with HIV: a qualitative study at a district antiretroviral therapy clinic in the Ashanti Region, Ghana
Source: Front Reprod Health. 2026 Sep 29;8:1914707. doi: 10.3389/frph.2026.1914707 (PMC13658555; doi:10.3389/frph.2026.1914707)
Supplement: Supplementary file 2 [file Supplementaryfile2.docx]

**Supplementary Material S2**

**COREQ (Consolidated Criteria for Reporting Qualitative Research) Checklist**

**Manuscript:** Antiretroviral therapy, perceived health, and fertility decision-making among people living with HIV: a qualitative study at a district antiretroviral therapy clinic in the Ashanti Region, Ghana

**Authors:** Priscilla Asantewaa Boadi, Victor Luckyboy Dzramado

**Checklist source:** Tong A, Sainsbury P, Craig J. Consolidated criteria for reporting qualitative research (COREQ): a 32-item checklist for interviews and focus groups. Int J Qual Health Care. 2007;19(6):349–57. doi: 10.1093/intqhc/mzm042

**Version: 2.1. Supersedes versions 1.0 and 2.0. Version 2.0 revised items 16, 22, 26, 29, 30, 31, and 32 to reflect the narrowed Theme 1, the explicit statement on sample adequacy, and the updated theme counts. Version 2.1 updates all table cross-references to the table numbering used in the published article.**

**Note on completion.** Items are answered as performed, not as ideally performed. Where a COREQ item was not addressed in the original study, this is stated plainly rather than reconstructed. Six items fall into that category and are collected in the summary table at the end, with the corresponding limitation cited in the manuscript. Section numbers refer to the manuscript; page numbers should be inserted at proof stage.

**Domain 1: Research team and reflexivity**

**Personal characteristics**

| **No.** | **Item** | **Guide question** | **Reported response** | **Location** |
| --- | --- | --- | --- | --- |
| 1 | Interviewer or facilitator | Which author conducted the interview or focus group? | All twelve interviews were conducted by the first author (PAB), who also performed the initial coding. The second author (VLD) was not present at any interview and had no contact with participants. | Section 2.2 |
| 2 | Credentials | What were the researcher's credentials? | Registered nurse-midwife. Postgraduate (MPhil) candidate at the time of the study; the work formed her master's research. | Section 2.2 |
| 3 | Occupation | What was their occupation at the time of the study? | Nurse educator at a midwifery training college in Kumasi. Not employed by the study hospital and held no clinical post there. | Section 2.2 |
| 4 | Gender | Was the researcher male or female? | Female. The second author, who contributed to design, analytic discussion, and interpretation but not to data collection, is male. | Section 2.2 |
| 5 | Experience and training | What experience or training did the researcher have? | Training in qualitative interviewing received as part of her postgraduate programme. She had not previously conducted or published an independent qualitative study, which is relevant context for the depth achieved on some topics. | Sections 2.2, 4.6 |

**Relationship with participants**

| **No.** | **Item** | **Guide question** | **Reported response** | **Location** |
| --- | --- | --- | --- | --- |
| 6 | Relationship established | Was a relationship established prior to study commencement? | No. The interviewer had no prior relationship with any participant, held no clinical role in their care, and was not a member of the clinic team. Participants were introduced to her at the point of recruitment. | Section 2.2 |
| 7 | Participant knowledge of the interviewer | What did participants know about the researcher? | Participants were told she was a nurse and postgraduate student conducting research for an academic degree, that she was not part of the clinic team, and that nothing they said would be entered into their medical records or affect their treatment. The study purpose was explained during consent. | Sections 2.2, 2.8 |
| 8 | Interviewer characteristics | What characteristics were reported about the interviewer? Bias, assumptions, reasons and interests in the research topic. | Reported in full. Her nursing background led her to expect that clinical counselling would prove the dominant influence on reproductive thinking; reflexive journal entries record her revising this expectation when economic and relational considerations proved more prominent. Two power-related risks are stated: interviews took place inside the facility where participants receive care, which may have encouraged favourable accounts of services; and participants may have overstated adherence to a nurse interviewer, which is why the analysis reports perceived rather than verified adherence. | Section 2.2 |

**Domain 2: Study design**

**Theoretical framework**

| **No.** | **Item** | **Guide question** | **Reported response** | **Location** |
| --- | --- | --- | --- | --- |
| 9 | Methodological orientation and theory | What methodological orientation was stated to underpin the study? | Reflexive thematic analysis informed by an interpretive phenomenological orientation. Phenomenology guided the study's interest in participants' meaning-making; the procedure implemented was thematic. The study did not implement the idiographic, case-by-case sequence characteristic of interpretative phenomenological analysis and is not presented as IPA. The Theory of Planned Behaviour did not inform the interview guide or the coding frame; it was applied only at the interpretation stage, and its use is confined to the Discussion, where its limits are stated. | Sections 2.1, 2.7, 4.2 |

**Participant selection**

| **No.** | **Item** | **Guide question** | **Reported response** | **Location** |
| --- | --- | --- | --- | --- |
| 10 | Sampling | How were participants selected? | Purposive sampling. Variation was sought in sex, age, marital status, parity, employment, and education, and the achieved sample reflects that intention. Variation in partner HIV status was not systematically sought and was not consistently recorded, which is reported as a limitation. | Sections 2.4, 4.6 |
| 11 | Method of approach | How were participants approached? | Face to face during routine clinic visits. A senior clinic nurse who was not a member of the research team identified eligible patients from the appointment list and asked whether they were willing to hear about the study; those who agreed were introduced to the interviewer in a private room. The manuscript states that this route favours patients engaged in care and comfortable with clinic staff, so patients disengaged from care or dissatisfied with services are likely underrepresented. Section 4.3 notes that a clinic-based route in a facility whose reproductive services are oriented toward women will tend to reproduce the documented underrepresentation of men in HIV care. | Sections 2.4, 4.3, 4.6 |
| 12 | Sample size | How many participants were in the study? | Twelve were interviewed: nine women and three men. The material available for four participants did not speak substantively to any reported theme, so the effective analytic sample was eight. Both figures are reported. | Sections 2.4, 3.1, 3.6; Tables 1, 2 |
| 13 | Non-participation | How many people refused to participate or dropped out? Reasons? | **Not documented.** Formal counts of patients approached, assessed as eligible, declining, and enrolled were not maintained during fieldwork. Reported openly as a documentation shortcoming and not reconstructed retrospectively. | Sections 2.4, 4.6 |

**Setting**

| **No.** | **Item** | **Guide question** | **Reported response** | **Location** |
| --- | --- | --- | --- | --- |
| 14 | Setting of data collection | Where was the data collected? | A private room at the antiretroviral therapy clinic of St. Michael's Hospital, Pramso, Bosomtwe District, Ashanti Region. The facility is a district-level Catholic mission hospital within the Christian Health Association of Ghana network, described because of its potential bearing on reproductive counselling. | Sections 2.3, 2.5, 4.4 |
| 15 | Presence of non-participants | Was anyone else present besides the participants and researchers? | No. Each interview was one to one with no third party present. For participants with limited literacy an impartial witness was present at the consent stage only, not during the interview. | Sections 2.5, 2.8 |
| 16 | Description of sample | What are the important characteristics of the sample? | Reported in Table 1: participant code, age band (five-year bands), sex, marital status, education, employment, number of children, and children's HIV status. Children's HIV status is identified as participant-reported and not verified against clinical records. Partner HIV status is not tabulated because it was not systematically recorded. Table 2 additionally reports which participants contributed to each theme, so that the composition of the analytic sample is visible alongside the demographic sample. Data were collected between 4 August and 5 October 2024. | Sections 3.1, 3.6; Tables 1, 2 |

**Data collection**

| **No.** | **Item** | **Guide question** | **Reported response** | **Location** |
| --- | --- | --- | --- | --- |
| 17 | Interview guide | Were questions, prompts, guides provided by the authors? Was it pilot tested? | The guide's full content is reported: time since diagnosis, experience of living with HIV, current treatment status, experience of health services, existing children and their HIV status, current thoughts about having more children, reasons for wanting or not wanting children, the influence of HIV status on those thoughts, factors considered most important, how providers addressed fertility concerns, counselling received, barriers encountered, and how those barriers affected decision-making. Probes invited elaboration and examples. The guide was refined with the academic supervisor but **not formally pilot tested**. Two boundaries are stated: it did not ask about viral load results, undetectable status, or viral load monitoring; and it did not ask separately about contraception, child spacing, or partner participation in decisions. Section 4.2 additionally notes that the guide asked about health and about children separately rather than about the relationship between them, which bears on the interpretation of Theme 1. | Sections 2.5, 4.1, 4.2, 4.3 |
| 18 | Repeat interviews | Were repeat interviews carried out? If yes, how many? | No. Each participant was interviewed once. | Sections 2.5, 2.9 |
| 19 | Audio or visual recording | Did the research use audio or visual recording to collect the data? | Audio recording was used with participants' permission. No visual recording was made. | Section 2.5 |
| 20 | Field notes | Were field notes made during and after the interview? | Yes. Field notes recorded setting, participant demeanour, and interviewer impressions. Their analytic use is specified: they contextualised transcript readings and informed reflexive journaling. They are not treated as an independent data source, and the term triangulation is not applied to them, since notes and transcript derive from the same encounter. | Sections 2.5, 2.9 |
| 21 | Duration | What was the duration of the interviews? | Between 45 and 90 minutes. | Section 2.5 |
| 22 | Data saturation | Was data saturation discussed? | Yes, and explicitly qualified. The interviewer and academic supervisor jointly judged, after twelve interviews, that later interviews were echoing earlier ones on three core topics (treatment access, transmission concern, counselling experience) without generating new categories. This is reported as a contemporaneous judgement about code-level adequacy on those topics, **not** as demonstrated saturation. Two features of the achieved dataset qualify it further and are stated in the manuscript: the effective analytic sample was eight rather than twelve, and substantive fertility-related content was available from only one of the three men. The manuscript therefore states that saturation cannot be claimed for the dataset as a whole, and cannot be claimed at all for men's accounts or for the mechanics of fertility decision-making. Information power is invoked as a framework applied **retrospectively** to appraise the achieved sample; the manuscript states that it did not guide the original sampling strategy and did not inform the decision to stop recruiting. | Sections 2.4, 4.6 |
| 23 | Transcripts returned | Were transcripts returned to participants for comment or correction? | **No.** Transcripts were not returned to participants. | Section 2.9 |

**Domain 3: Analysis and findings**

**Data analysis**

| **No.** | **Item** | **Guide question** | **Reported response** | **Location** |
| --- | --- | --- | --- | --- |
| 24 | Number of data coders | How many data coders coded the data? | Two. The first author coded all transcripts. A second coder with qualitative experience independently coded a subset, and discrepancies were resolved by discussion. | Sections 2.7, 2.9 |
| 25 | Description of the coding tree | Did authors provide a description of the coding tree? | Yes. The full codebook of 23 inductive codes and the mapping of codes to sub-themes and themes are provided in Supplementary Material S1. The analytic hierarchy is defined explicitly: codes describe segments of talk, sub-themes group codes addressing one aspect of experience, and themes group sub-themes. | Section 2.7; Supplementary S1 |
| 26 | Derivation of themes | Were themes identified in advance or derived from the data? | Derived from the data. Coding was inductive, generated from participants' own language with no prior coding frame and no theoretical categories imposed. Candidate sub-themes that a full check against the transcripts did not support were removed rather than retained on thin evidence, and the four themes reported are those the data sustain. Theme 1 was subsequently renamed from a formulation referencing perceived bodily health to "Treatment access and service appraisal," because a participant-by-participant check established that only one of its four contributing accounts linked medication-taking to felt wellness, and that no account connected wellness to childbearing. Its sub-theme counts are reported separately in Table 3 for this reason. | Sections 2.7, 3.2, 3.3; Table 3 |
| 27 | Software | What software was used to manage the data? | **None.** Coding was performed manually, using printed transcripts, marginal annotation, and a spreadsheet register of codes. | Section 2.7 |
| 28 | Participant checking | Did participants provide feedback on the findings? | **No.** The interviewer summarised and checked her understanding with participants during interviews, which is reported as in-interview clarification and explicitly distinguished from member checking. Transcripts were not returned, no repeat interviews were held, and participants did not review the findings. | Section 2.9 |

**Reporting**

| **No.** | **Item** | **Guide question** | **Reported response** | **Location** |
| --- | --- | --- | --- | --- |
| 29 | Quotations presented | Were participant quotations presented to illustrate the themes? Was each quotation identified? | Yes. Quotations appear in manuscript Tables 4 to 7 and in Supplementary Material S1. Each is identified by participant code (P1 to P12) with a de-identified descriptor giving sex, age band, marital status, and parity. Every quotation and participant identifier was verified against the original audio recordings, and code and sub-theme assignments were checked against the utterances they were drawn from; the verification record, including corrections to attributions carried from the underlying thesis, is documented in Supplementary Material S1. Composite quotations joining non-contiguous speech have been dismantled, so each excerpt is contiguous speech from a single answer unless an ellipsis marks omission. Where an excerpt was given in answer to a specific question, this is stated, so that reported counselling content is distinguishable from unprompted participant reflection. | Section 2.9; Tables 4–7; Supplementary S1 |
| 30 | Data and findings consistent | Was there consistency between the data presented and the findings? | Addressed directly. Each excerpt table carries a column confined to what the quotation establishes, kept separate from interpretation, which is reserved for the surrounding text and Discussion. In Table 4 this column now records, participant by participant, that one excerpt links medication-taking to felt wellness without connecting it to childbearing, that one concerns pharmacological safety rather than the participant's own health, and that two are service appraisals carrying no reproductive content. Quantifying language is avoided in favour of explicit counts traceable to Table 2. Claims about viral suppression are absent, since no participant referred to viral load, undetectable status, or monitoring. Section 4.2 reports the treatment-to-reproduction link as one that regional literature predicts but that these data do not demonstrate, and offers two candidate explanations without selecting between them. | Sections 3.3–3.6, 4.1, 4.2; Tables 2, 4–7 |
| 31 | Clarity of major themes | Were major themes clearly presented in the findings? | Yes. Four themes are presented, each in its own Results subsection with an excerpt table and accompanying account: treatment access and service appraisal (4 participants); transmission concern and reported prevention information (6); reproductive counselling as experienced by patients (5); and heterogeneity of fertility positions (8). Contributing-participant counts are displayed with each theme, and the manuscript states that they record contribution of material rather than agreement. Theme 1 additionally reports sub-theme-level counts (access and adequate care, 4; medication-to-wellness link, 1; medication safety in pregnancy, 1) so that its uneven evidential base is visible in the table itself. The manuscript states that no theme rests on the whole sample and that Theme 1's basis for anything beyond access and satisfaction is a single account. | Sections 3.2–3.6; Tables 2, 3; Figure 1 |
| 32 | Clarity of minor themes | Is there a description of diverse cases or discussion of minor themes? | Yes. Theme 4 reports heterogeneity of reproductive positions as a finding in its own right, distinguishing deferral, aspiration held with uncertainty, limitation after a completed family, and settled non-intention. Divergent and negative cases are tabulated separately in Supplementary Material S1 and reported in the manuscript, including the male participant who explicitly denied that his HIV status influenced his reproductive thinking and framed the decision economically, the participant who reported not having received fertility-specific counselling, and the participant who received counselling and disregarded it. The single account naming partner pressure is reported and explicitly marked as a single instance. Material excluded from analysis, and the reasons for exclusion, is reported in Supplementary Material S1. | Sections 3.5, 3.6; Table 5; Supplementary S1 |

**Summary of items not fully met**

| **Item** | **Shortfall** | **Where acknowledged** |
| --- | --- | --- |
| 13. Non-participation | Counts of those approached, eligible, declining, and enrolled were not maintained | Sections 2.4, 4.6 |
| 17. Interview guide | Not formally pilot tested; did not ask about viral load, contraception, spacing, or partner involvement; asked about health and about children separately rather than about the relationship between them | Sections 2.5, 4.1, 4.2, 4.3, 4.6 |
| 22. Data saturation | Stopping decision was a contemporaneous code-level judgement on three topics, not demonstrated saturation; effective analytic sample was eight; substantive male content from one participant; information power applied retrospectively | Sections 2.4, 4.6 |
| 23. Transcripts returned | Transcripts were not returned to participants | Section 2.9 |
| 28. Participant checking | No member checking; in-interview clarification only | Section 2.9 |
| Language and translation (not a numbered COREQ item, reported for completeness) | Twi recordings transcribed and translated in a single step by one bilingual researcher; no independent back-translation | Sections 2.6, 4.6 |
